# The Application of a Meiocyte-Specific CRISPR/Cas9 (MSC) System and a Suicide-MSC System in Generating Inheritable and Stable Mutations in Arabidopsis

**DOI:** 10.3389/fpls.2018.01007

**Published:** 2018-07-13

**Authors:** Penghui Xu, Hang Su, Wanli Chen, Pingli Lu

**Affiliations:** State Key Laboratory of Genetic Engineering, Ministry of Education Key Laboratory of Biodiversity and Ecological Engineering, Institute of Plant Biology, School of Life Sciences, Fudan University, Shanghai, China

**Keywords:** meiocyte-specific promoter, CRISPR/Cas9, genome editing, suicide-MSC, Arabidopsis

## Abstract

The CRISPR/Cas9 system has been widely used for generating targeted mutations in various species. In Arabidopsis, it largely relies on the edited cells where the Cas9 protein performs its activity to obtain heritable and stable mutated lines. Here, we designed an improved CRISPR/Cas9 system, named as the MSC (meiocyte-specific CRISPR/Cas9) system, in which the *Cas9* expression is driven by an experimentally approved meiocyte-specific promoter (*AtDMC1* promoter). Two endogenous genes, including vegetative gene *AtDET2* and reproductive gene *AtDMC1*, were targeted. We obtained heterozygous T1 plants for targeted genes with high efficiency (64%). In the T2 generation, the homozygous plants were abundant with high efficiency (37%). Analysis of Sanger sequencing results of T2 generation revealed that heritable gene mutations were high (52%). Moreover, we showed that the MSC system could sufficiently delete a middle size DNA fragment (∼500 bp) between two cleavage sites with a high rate (64.15%) in the T1 plants, providing direct evidence for making complete knock-out or certain domain-depletion mutations. In addition, we further made a suicide-MSC system, which can edit the targeted endogenous gene and the exogenous *Cas9* gene simultaneously, not only successfully avoiding the further destroy of alleles brought in by molecular complementary or genic allelic test, but also maintaining the stable mutated alleles for functional studies. In short, the two systems provide new approaches to generate mutations for gene functional studies.

## Introduction

In the past decades, genetic studies, especially based on mutant analyses, have greatly enriched our knowledge about gene functions in various biological processes. To generate more mutations, scientists have used multiple approaches to satisfy their studies and made generous contributions to research communities. Physical and chemical agents have been extensively used to randomly generate mutants with a large population (Page and Grossniklaus, 2002). As for Arabidopsis, due to the easiness of genetic transformation via *Agrobacterium*-mediated T-DNA insertion, a large collection of mutant lines has been made and available for researchers, offering a gene-indexed resource for reverse genetic studies at a whole genome scale (Alonso et al., 2003). However, there are still about 12% of genes without T-DNA insertion lines available (O’Malley and Ecker, 2010). Although the RNA interference (RNAi) approach is an alternative option for loss-of-function studies for the above-mentioned genes (Alonso and Ecker, 2006), the interference levels are greatly variable among different transgenic lines, and even the stability of mRNA silencing efficiency is changing during generation to generation.

To make mutations for targeted genes, different engineered endonuclease systems have been developed in distinct species, such as zinc-finger nucleases (ZFNs), transcription activator-like effector nucleases (TALENs), and the clustered regularly interspaced short palindromic repeats (CRISPR)/CRIPR-associated protein9 (Cas9) endonuclease (CRISPR/Cas9) systems (Kim et al., 1996; Miller et al., 2011; Sternberg et al., 2014). Compared to ZFNs and TALENS systems, the CRISPR/Cas9 is relatively simple. It contains two major components, a single-guide RNA (sgRNA) (recognizing the targeted sequence based on RNA-DNA base pairing) and the CRISPR/Cas9 endonuclease (generating DNA double strand breaks; DSBs). The DSBs are thought to be repaired via the non-homologous end-joining (NHEJ) pathway to produce insertion/deletion mutations at the target sites (Sander and Joung, 2014). Due to its easier use, the CRISPR/Cas9 system has been widely used in many species to obtain targeted gene mutations (Cong et al., 2013; Xing et al., 2014; Gao et al., 2017; Puchta, 2017).

In plants, to obtain genome edited mutations, the CRISPR/Cas9 system is usually delivered to cells via *Agrobacterium*-mediated transformation, resulting in the integration of the CRISPR/Cas9 cassette in the genome. The expression of *Cas9* is highly associated with the efficiency of targeted mutagenesis. In most cases, the cauliflower mosaic virus *35S* promoter was employed to drive *Cas9* expression (Fauser et al., 2014; Feng et al., 2014). However, the efficiency of targeted mutagenesis is very low, potentially due to the weak activity of the *35S* promoter in reproductive cells (Fauser et al., 2014). To overcome the shortage, several other promoters were used to drive *Cas9* expression in Arabidopsis, aiming to increase the efficiency of genome editing. For example, the *YAO* promoter, which is preferentially active in the tissue undergoing cell division, is shown to work better (Yan et al., 2015). Furthermore, germ-line-specific promoters, such as the *SPOROCYTELESS* promoter and the egg cell-specific *EC1.2* promoter, were used to improve the efficiency of targeted gene mutagenesis (Wang et al., 2015; Mao et al., 2016). However, those promoters are working before or after meiosis, in which diploid reproductive cells undergo meiosis to produce haploid daughter cells. Moreover, the presence of the CRISPR/Cas9 system in the edited mutant genome will affect the subsequently molecular complementary experiment or allelic test by genetic cross, because the CRISPR/Cas9 system will recognize the wild type allelic sequence brought in to destroy it. Although CRISPR/Cas9-free mutants can be obtained by screening a next generation population from a hybrid plant generated by genetic cross with wild type, sometimes it is extremely difficult to get ideal mutants due to closely genetic linkages. Therefore, it is also necessary to make a system, in which the CRISPR/Cas9 system can be destroyed after the targeted gene is edited.

Here, we developed a meiocyte-specific CRISPR/Cas9 (MSC) system, in which the expression of *Cas9* is driven via a meiocyte-specific promoter (*AtDMC1* promoter) in Arabidopsis. Two genes, including *AtDET2* (*AT2G38050*) and *AtDMC1* (*AT3G22880*), which have not been selected as tested genes in previously reported Cas9-mediated genome editing studies, were used as targeted genes. Our data showed that the system can greatly generate heritable mutations in T2 population. Furthermore, we developed a suicide CRISPR/Cas9 system based on the MSC system, termed as suicide-MSC system, showing that it not only can generate targeted mutations, but also can efficiently destroy the *Cas9* gene, simultaneously. The suicide-MSC system will greatly facilitate the molecular complementary and genetic allelic test studies for confirming the targeted gene function.

## Results

### Design of a Meiocyte-Specific CRISPR/Cas9 System for Genome Editing

In Arabidopsis, although more than 90 genes have been discovered to be involved in meiosis (Wang and Copenhaver, 2018), the mRNAs of most those genes are broadly expressed in various tissues. However, the expression of the *AtDMC1* gene, which encodes a meiocyte-specific recombinase, was shown to be highly restricted to meiocytes by both RNA *in situ* hybridization analysis and promoter activity test with the *GUS* reporter gene (Klimyuk and Jones, 1997), providing a great opportunity to generate a MSC system. Genetics and cytological analyses revealed that *AtDMC1* is involved in meiotic recombination, facilitating the meiotic crossover formation during meiosis I (Couteau et al., 1999). In this study, we planned to use an *AtDMC1* promoter driving the *Cas9* expression to achieve a MSC system in Arabidopsis. First, we cloned the previously reported *AtDMC1* promoter, containing a 3.1 kb long genomic fragment upstream of the ATG start coding, from L*er* wild type background genome. The resulting *AtDMC1* module was then cloned into the pRGEB31 binary vector (Supplementary Figure S1B) (Xie et al., 2015) to replace the original Pro:*35S* fragment, leading to the formation of MSC plasmid (pMSC) (Supplementary Figure S1A). Hereafter, we designated the plasmid as the MSC system. The resulting pMSC vector allows one-step ligation of multiple annealed 20-nt oligos for target recognition at the 2 ×*Bsa* I sites. For testing the MSC system, two protein-coding genes, *AtDET2* (*AT2G38050*) and *AtDMC1* (*AT3G22880*) were targeted, respectively.

### Targeted Mutations of *AtDET2* by the MSC System

**FIGURE 1:**
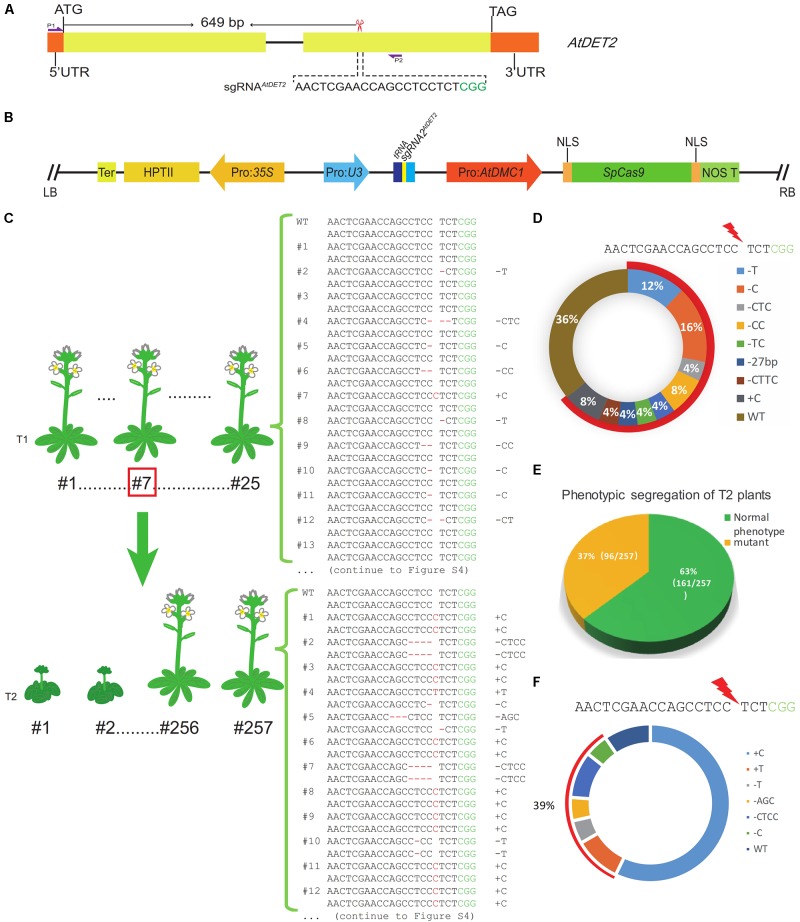
Efficient targeted mutagenesis for the *AtDET2* gene edited by the MSC system in Arabidopsis. **(A)** The genomic targeted site in the *AtDET2* gene. The sgRNA*^AtDET2^* sequence is shown in black and the green sequence is the PAM (protospacer adjacent motif). The red scissor indicates the position of the targeted site. P1 and P2: a pair of specific primers for amplifying the DNA fragment that covers the target site. **(B)** A structural representation of the pLFC286 plasmid, showing the SpCas9 endonuclease driven by Pro: *AtDMC1*; and the sgRNA*^AtDET2^* driven by the Pol-III promoter *U3*. LB/RB, T-DNA left/right border; NOS T, *nos* gene terminator; HPTII, hygromycin-resistance gene; Ter, terminator. **(C)** Genotyping results of *Atdet2* mutants generated by the MSC system in the T1 and T2 generations. The relationship between generations was shown in the panel. 25 T1 transgenic lines and 23 T2 lines were sequenced. The genotypes were listed on the right. **(D)** Genotypes and their relative frequency of T1 transgenic plants. Aligning with the wild-type sequence, 36% plants with no change in sequence. However, 64% plants are heterozygous. The 20 bp black sequence is sgRNA*^AtDET2^* and the green sequence is PAM, red lightning symbol represents the DSB formation site. **(E)** Phenotypic segregation analysis for the T2 plants. Plants with a normal like phenotype account for 63% (161/257). Plants displaying *Atdet2* mutant phenotypes occupy 37% (96/257). **(F)** Genotypes and their relative frequency of T2 plants. Aligning with the wild-type sequence, 39% targeted mutations are *de novo* mutations that aren’t inherited from parental plant. 9% plants were no change in sequence and the genotype with 1 bp C insertion (+C) account for 52%, which are mostly inherited from the parental plant #7. The black sequence is sgRNA*^AtDET2^* and the green letter is PAM.

In order to more conveniently test the efficiency of the MSC system, we preferred to select a targeted gene whose mutants display easily observed phenotypes. In Arabidopsis, the *AtDET2* gene, encoding a functional Steroid 5α-Reductase, was involved in Brassinosteroid (BR) biosynthesis (Li et al., 1997). The mutants of *AtDET2* displayed dark-grown seedlings with curly leaves, resulting in dwarf and a prolonged vegetative phase (Chory et al., 1991). Those phenotypic defects greatly satisfied our above criteria. Therefore, we selected *AtDET2* as the first targeted gene for testing. We used the online tool^1^ to design the sgRNA (single guide RNA) for *AtDET2* and subsequently named it as sgRNA1*^AtDET2^*. The location and sequence of the sgRNA1*^AtDET2^* were shown in the **Figure 1A**. In the MSC system, the *PTGs* (Polycistronic tRNA-gRNAs) were synthesized by GG (Golden Gate) assembly (Engler et al., 2008). The tRNA-gRNA1*^AtDET2^* fragment, which was designated as *PTG1* (Supplementary Table S2), was cloned and inserted into the pMSC plasmid, forming the pLFC286 (**Figure 1B**) (seen details in the section “Materials and Methods”). *Agrobacterium*-mediated transformation was carried out in Arabidopsis further.

For this CRISPR/Cas9 binary construct, we totally obtained 53 individual Arabidopsis transgenic T1 lines. In our data, we did not observe any T1 plants showing similar mutant defective phenotypes, suggesting that no *Atdet2* homozygous mutant was formed in the T1 generation. Subsequently, 25 individual lines were subjected to mutation detection by Sanger sequencing of PCR products (amplified with primers oLF1852 and oLF1853) surrounding the sgRNA targeted site from leaf tissue samples. Considering T1 edited plants are usually not homozygous, analyses were conducted by examining their sequencing chromatograms. We found that 16 transgenic lines were heterozygotes. However, the other nine lines were shown no changes in the sequenced data. Therefore, for the pMSC-gRNA1*^AtDET2^*, the heterozygosity rate is high with 64% (16/25). The genotypes of those plants are shown in the **Figure 1C** and Supplementary Figure S4. We found that the repaired outcome of the CRISPR/Cas9 DSBs (double strand breaks) induction via NHEJ pathway in the MSC system is highly variable. Totally eight distinct mutated genotypes were detected. However, for this specific targeted site on the genome, some NHEJ signatures were disclosed to be preferred over others, such as the 1 bp C deletion (16%) and the 1 bp T deletion (12%) (**Figure 1D**).

**FIGURE 2:**
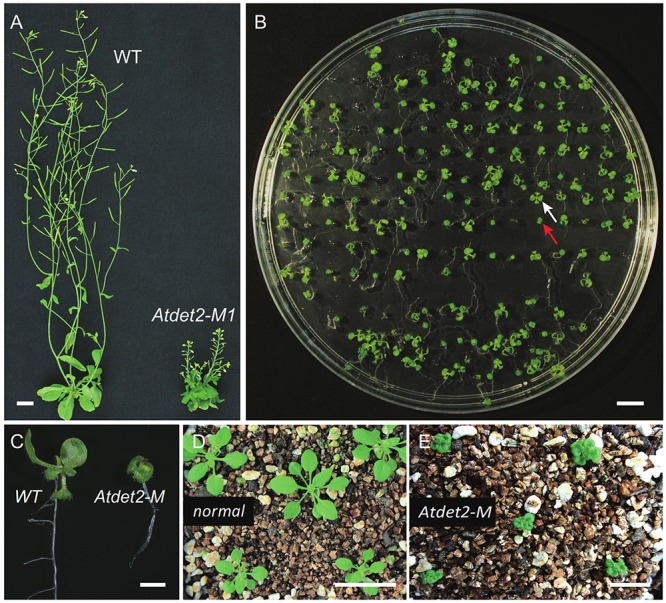
Phenotypic analyses of *Atdet2-M* mutants generated by the MSC system. **(A)** Phenotypes of wild type plant and targeted editing *Atdet2* mutant plant. Both have grown for 45 days before photographing. Bar = 1 cm. **(B)** Phenotypic segregation of T2 plants. The white arrow points a seedling with normal like phenotype, and the red arrow points an *Atdet2* mutant seedling with small and dark-grown leaves. Plants have grown for 12 days on 1/2 Murashige and Skoog (MS) medium before photographing. Bar = 1 cm. **(C)** Phenotypes of wild type plant and targeted editing *Atdet2* mutant plant. Plants have grown on 1/2 MS medium for 12 days. Bar = 1 mm. **(D)** Phenotypes of normal like plants grown for 20 days in soil. Bar = 1 cm. **(E)** Phenotypes of *Atdet2* mutants grown for 20 days in soil. Bar = 1 cm.

To comprehensively investigate the mutagenesis efficiency of the MSC system at the targeted gene in the T2 generation, we analyzed the phenotypes of 257 individual T2 plants (Supplementary Table S4), which were generated from the T1 line #7. The T1 line #7 is heterozygote genotype with 1 bp C insertion (+C), showing a normal-like phenotype (Supplementary Figure S2A). Sequencing chromatogram was shown in the Supplementary Figure S2B. In the T2 generation, phenotypic separation was observed (**Figure 2A**). We found that 96 individual plants showed similar phenotypic defects to the *Atdet2* mutant’s (**Figures 2B,C**), indicating the homozygosity rate is very high with 37% (96/257) in the T2 generation (**Figure 1E**). Theoretically, considering the line #7 is heterozygous, it is expected to see ∼25% plants showing mutant phenotypes based on the Mendel genetic law. However, the actually observed value of 37% is much larger, implying that the MSC system further edited the targeted gene in the T2 generation. To investigate in more detail the number and nature of the gene mutations present in the MSC-generated T2 populations, we selected 5 normal-like and 18 mutant phenotypic plants for sequencing genotypic analysis (**Figures 2D,E**).

The sequencing results revealed that all plants displaying mutant phenotypes (line #1∼18) were homozygous or biallelic at the targeted sites. While those normal-like plants (line #50∼54) were detected to be all heterozygous. Therefore, homozygous plants accounted for a higher proportion (79%, 18/23) and heterozygous plants occupied 21% (5/23) (**Table 1**). Subsequently, we analyzed the sequencing chromatograms and compared it with the parental genotypes, finding that the mutant allele with 1 bp C insertion (+C) accounted for 52%. That means the gametes with +C genotype occupied 52% of the total number of gametes. Given that the genotype of line #7 is heterozygous with 1 bp C insertion (+C), theoretically the ratio of the +C genotypic gametes is expected to be 50% in the gametes population. However, the observed data (52%) is higher than the theoretical one (50%). We reasoned that some gametes with +C genotype are newly generated by the remained MSC system. Consistently, we also found other types for the genome editing, including 4 bp CTCC deletion (-CTCC), 1 bp T insertion (+T), and 3 bp AGC deletion (-AGC). The ratio of newly generated mutations is ∼39% (**Figure 1F**). To further confirm the genome editing efficiency in the T2 generation, we investigated T2 progenies from two other T1 lines (#19 and #33) with heterozygous mutated genotypes. Our data showed that in the progenies of T1 lines #19 and #33, the homozygous or biallelic plants accounted for 29% and 57%, respectively. These results further proved that the MSC system can generate higher mutation rate in the T2 generation (Supplementary Figure S5).

**Table 1:** Mutation analysis of T1 and T2 plants.

		Positive transgenic plants	Number of sequencing	Number of heterozygotes	Percentage of heterozygotes	Number of homozygotes	Percentage of homozygotes
sgRNA^At^*^DET2^*	T1	53	25	16	64%		
	T2		23	5	21%	18	79%
sgRNA^At^*^DMC1^*	T1	27	13	9	69%		
	T2		7	1	14%	6	86%

### Targeted Mutation of Meiotic Gene *AtDMC1* by the MSC System

**FIGURE 3:**
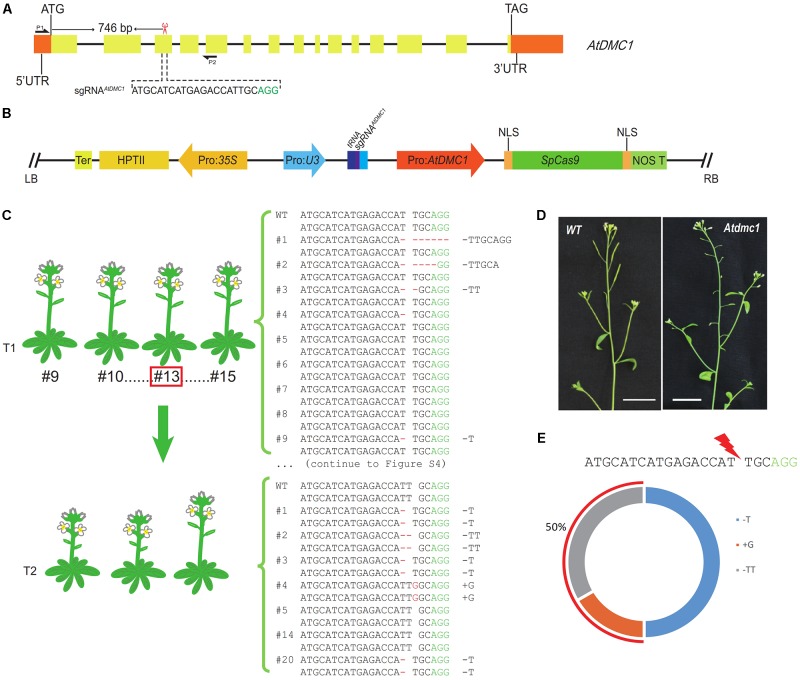
Genotypic and phenotypic analyses of targeted mutations generated by the MSC system for *AtDMC1* in Arabidopsis. **(A)** The targeted site in the *AtDMC1* genomic region. The sgRNA*^AtDMC1^* was located in the third exon (746 bp downstream of the start codon). The sgRNA*^AtDMC1^* sequence was shown in black and PAM in green. The red scissor indicates the targeted position. P1 and P2 showed the primers used for amplifying the DNA fragment covering the targeted site. **(B)** The map of pLFC290 plasmid. An illustration of the SpCas9 endonuclease driven by Pro: *AtDMC1*, and the sgRNA*^AtDMC1^* driven by Pol-III promoters *U3*. **(C)** Sanger sequencing results of *Atdmc1* mutants generated by the MSC system in T1 and T2 generation. In the T1 generation, heterozygosity rate is 69% (9/13). The analyzed T2 population was derived from the parent line #13 (framed by a red square). Heterozygosity rate is 86% (6/7) in the T2 generation. **(D)** The phenotypes of the wild type plant and the targeted editing *Atdmc1* mutant in T2 population. The mutant’s siliques are shorter than wild-type ones, suggesting the fertility is affected. Bar = 1 cm. **(E)** The genotypic statistics of T2 plants. 50% of the targeted editing mutations are newly mutations. Another 50% were inherited from the parental plant line #13 (+G).

To further test mutagenesis efficiency of the MSC system, we selected another targeted gene. As most previously tested genes in CRISPR/Cas9 mediated systems are restricted to vegetative functional genes, because its mutant phenotypes can be observed during early developmental stages. However, this strategy might limit the efficient test due to genomic location variation and developmental influence. Here, we selected a meiotic gene named *AtDMC1*, which is involved in meiotic recombination (Couteau et al., 1999), to test the efficiency of the MSC system in reproductive genes. Similar to the previous construction, the sgRNA for *AtDMC1* (named as sgRNA*^AtDMC1^*) was designed via the online tool (see footnote 1). The location and sequence of the sgRNA1*^AtDMC1^* were shown in the **Figure 3A**. The fragment *PTG2* (Supplementary Table S2) containing sgRNA*^AtDMC1^* was cloned and inserted into the pMSC plasmid, leading to final plasmid pLFC290 (**Figure 3B**) (seen details in the section “Materials and Methods”).

For this CRISPR/Cas9 binary vector for Arabidopsis transformation, we totally gained 27 T1 transgenic lines. However, we did not detect any T1 plant showing *Atdmc1* mutant phenotypes, like short siliques and pollen fertility defects. This result suggested that there is no homozygous or biallelic genotypes in the T1 plants. This result is consistent with the data obtained from *AtDET2* editing case. To investigate the genome edited outcomes for the targeted site, we randomly selected 13 individual transgenic T1 lines subjected to mutation detection by Sanger sequencing. We found that nine T1 lines were heterozygous; and the other four lines showed no changes at the sgRNA*^AtDMC1^* target site in alignment with the wild-type sequence. All genotypes of those lines were shown in the **Figure 3C** and Supplementary Figure S4. Thus, we concluded that the heterozygosity rate generated in the T1 population is reached to 69% (9/13) high.

In order to evaluate the efficiency of the MSC-sgRNA*^AtDMC1^* system in the next generation, we selected the T1 line #13 with heterozygous genotype (+G) as parental to test its T2 progenies. In a T2 population including 65 individuals, as expected, plants showing similar phenotypes with *Atdmc1* mutant (very short siliques and reduced fertility) were observed (**Figure 3D**). However, the number of plant with mutant phenotype is 22, occupying 34% of total examined plants. This number is significantly higher than that in theoretically produced by Mendel law of segregation for a heterozygous parental plant, meaning new genetic mutations were generated. To detect the mutated types in the T2 population, we selected seven individual lines subjected to mutation detection by Sanger sequencing, finding that six lines were homozygous for the targeted site (**Table 1**). It was revealed that the +G mutation, which was present in the parental genome, accounted for 50%, in agreement with the expected data from genetics. However, the other 50% mutated genotypes, including 1 bp T deletion (-T), 1 bp G insertion (+G) and 2 bp TT deletion (-TT) (**Figure 3E**), were newly generated in the T2 generation, showing a higher efficiency of edited alleles.

### Efficiently Generating Middle Size Deletions Between Two Cleavage Sites by the MSC System

**FIGURE 4:**
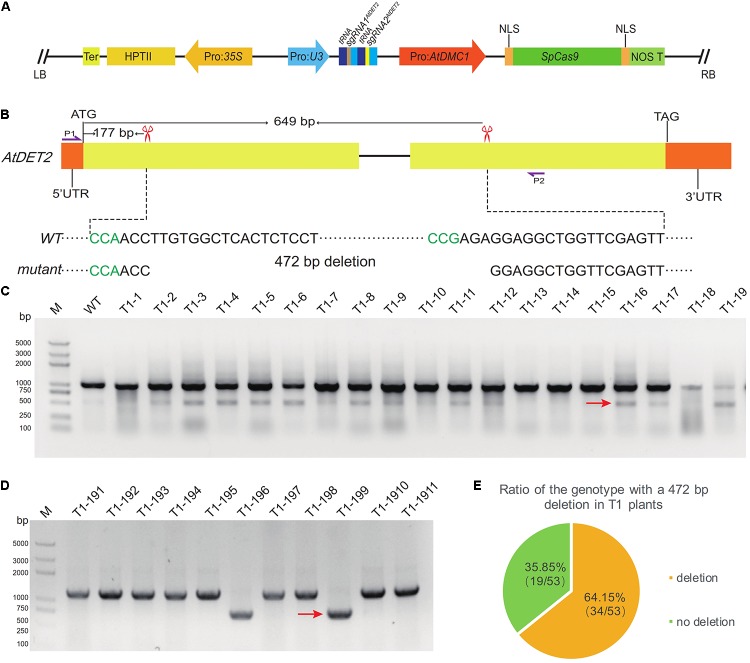
Efficient generation of a middle size deletion by the MSC system. **(A)** A structural representation of the pLFC317 plasmid. **(B)** Two targeted sites were designed to expectedly generate a 472 bp deletion in the *AtDET2* gene. The red scissors indicated the targeted sites of *AtDET2*. The left black sequence is sgRNA1*^AtDET2^* and the right is sgRNA2*^AtDET2^*. The PAM sequence is shown in green. The purple arrows P1 and P2 represent the locations of primers for amplifying the DNA fragment embracing the two cleavage sites. **(C)** PCR detection of DNA deletion between the two targeted sites in the T1 transgenic plants. Successful deletions were shown as shorter PCR products (indicated with red arrow). **(D)** The deletion was indicated by the results of colony PCR. The red arrow represents PCR products with deletion. **(E)** The deletion frequency in T1 transgenic plants.

To evaluate the efficiency of simultaneous editing at two targeted sites, expecting to produce a designed deletion, by the MSC system, we selected two targeted sites in the *AtDET2* genomic region, named as sgRNA1*^AtDET2^* and sgRNA2*^AtDET2^*, for direct test. The detailed information of sequences and locations for the two sgRNAs were shown in the **Figure 4B**. To make the CRISPR/Cas9 construct, the *PTG3* (Supplementary Table S2) harbored the sgRNAs was synthesized by the GG assembly. Subsequently, the *PTG3* fragment digested by *Fok* I was cloned into the pMSC vector, giving rise the final plasmid named pLFC371 (**Figure 4A**). For this CRISPR/Cas9 binary construct, we totally obtained 53 T1 individual transgenic lines. Genomic DNA was extracted from T1 plants grown in soil. To analyze the mutated types of *AtDET2* in the T1 plants, we amplified a fragment spanning the two target sites by the PCR approach, using a pair of gene-specific primers (oLF1852 and oLF1853). Then the amplified products were analyzed by Agarose Gel Electrophoresis. It was revealed two expected bands were observed in T1 lines (**Figure 4C**), suggesting that the designed system indeed generated the expected deletion in the targeted region. To further confirm that, the amplified smaller band was analyzed by colony sequencing (**Figure 4D**). We found that the 472 bp fragment between two sgRNAs cleavage sites was excised from the targeted loci with or without additional indels. Furthermore, we found that the frequency of designed deletion is relatively high in the T1 transgenic lines, showing 64.15% (34/53) by PCR genotyping (**Figure 4E**). Our results indicated that the MSC system mediated fragment deletion exhibited high efficiency at the studied sites. This system makes it possible to generate knock out mutations or mutations lost certain functional domains in genetics, consequently facilitating the molecular mechanism studies for gene functions.

### The Suicide-MSC System and Its Editing Efficiency

Although the CRISPR/Cas9 system is powerful for generating mutations at the targeted gene sites, the integration of the CRISPR/Cas9 cassette in the plant genome can make it difficult for the molecular complementary experiments for further gene functional confirmation, because the remained CRISPR/Cas9 system will work again to destroy the complementary allele. Although genetic crosses and following segregation could make it possible to obtain CRISPR/Cas9 cassette free mutants in the next generation, it usually takes longer time and costs more labor. Sometimes, when the T-DNA carrying the CRISPR/Cas9 cassette was inserted into the genomic location close to the targeted gene, it will be very hard to get CRISPR/Cas9 free edited mutants due to genetic linkage. Therefore, it is necessary to obtain edited mutants with destroyed CRISPR/Cas9 system in some cases, especially for those mutants showing reproductive defects.

**FIGURE 5:**
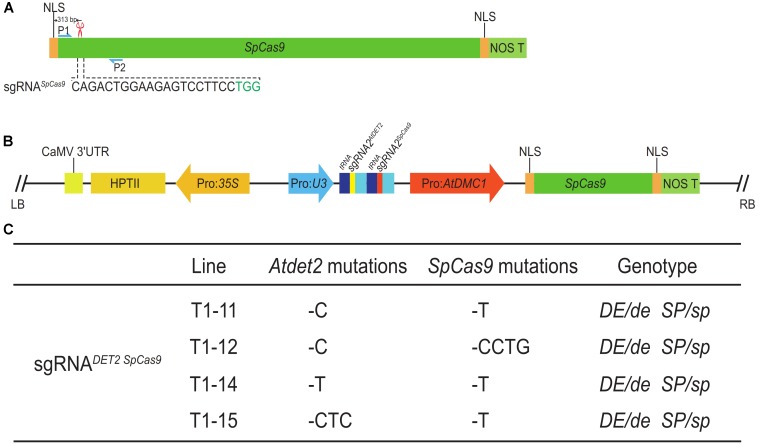
The construction of the suicide-MSC system and its editing efficiency. **(A)** Illustration of the targeted site for *SpCas9*. The sgRNA*^SpCas9^* was located on 313 bp downstream of the start codon. The sequence of sgRNA*^SpCas9^* was shown in black, and PAM was shown in green. The red scissor indicates the targeted site in *SpCas9*. The blue arrows represented the location of primers used for amplifying the DNA fragment for Sanger sequencing analysis. **(B)** The map of the pLFC312 binary vector, harboring two sgRNAs, sgRNA*^AtDET2^* and sgRNA*^SpCas9^*. **(C)** Sanger sequencing results revealed the genotypes of individual T1 lines with a normal phenotype. ‘–’ indicates deletion. *DE* and *SP* correspond to the wild-type allele of *AtDET2* and *SpCas9*. *de* and *sp* correspond to the mutated alleles of *AtDET2* and *SpCas9*.

Here, we designed a CRISPR/Cas9 system harboring a sgRNA for the *SpCas9* itself, named as sgRNA*^SpCas9^*, hoping to the CRISPR/Cas9 complex can also recognize and edit the *SpCas9* gene when the complex edit the targeted gene on the genome. The previous tested gene *AtDET2* was used again for the test. The sequences and locations of sgRNA*^SpCas9^* and sgRNA1*^AtDET2^* were shown in the **Figures 2A, 5A**, respectively. The T-DNA fragment of the final vector pLFC312 is shown in **Figure 5B**. After transformation of the Arabidopsis for this binary vector, we obtained six T1 transgenic lines. Similar to the above data for *AtDET2* alone, we did not observe any edited plants displaying *Atdet2* mutant phenotypes. To further investigate the genotypic changes for both *AtDET2* and *SpCas9* genes, we amplified the DNA fragments covering the targeted sites via specific primers for both genes, respectively. The sequencing results revealed that both the *SpCas9* and *AtDET2* genes were edited to form various mutations in four plants, with all being heterozygous (**Figure 5C**). The gene editing efficiency was as high as 67% (4/6) (Supplementary Table S3). Therefore, we concluded that the CRISPR/Cas9 system not only can make mutant for a targeted gene on the genome, but also can destroy itself at the same time. This suicide-MSC system makes it more convenient for genetic crosses (especially for allelic test) and molecular complement for CRISPR/Cas9 edited mutants.

## Discussion

### The Advantages of the MSC System in Generating Genome Editing Mutation

**FIGURE 6:**
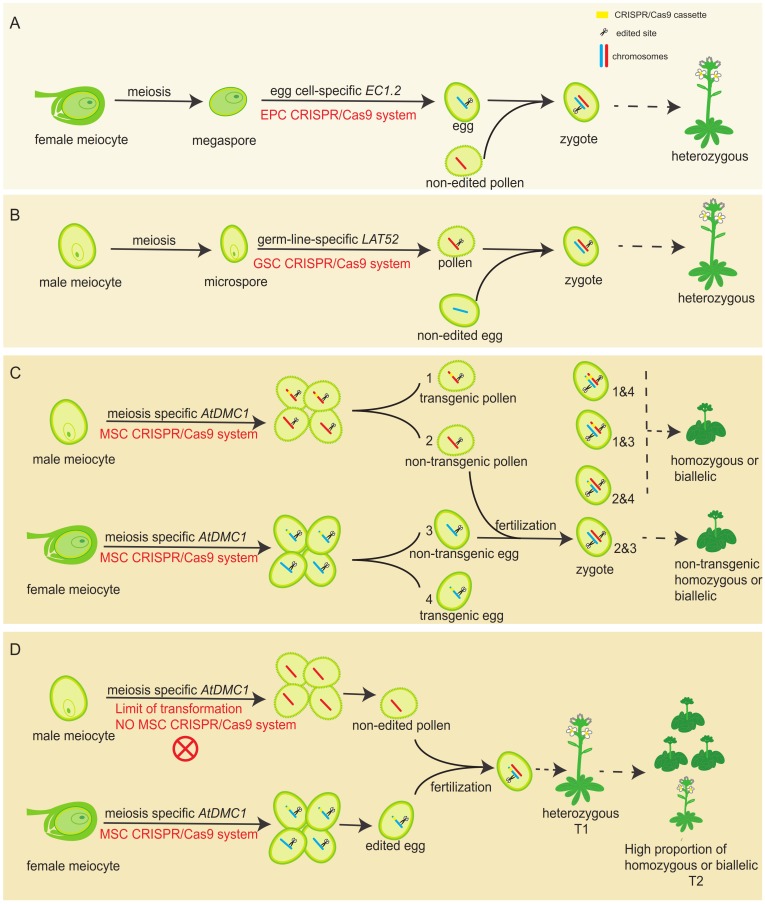
A comparison of germ-line specific promoters driving *Cas9* editing systems with the MSC system in generating edited mutations. **(A)** The working model for the egg-cell specific promoter-driving *Cas9* system. The editing system only performs its activity in female gamete side, leading to heterozygous plants in the T1 generation. **(B)** The working model for the pollen-specific promoter-driving *Cas9* system. The editing system only performs its activity in male gamete side, leading to heterozygous plants in the T1 generation. **(C)** The expected working model for the MSC system. The editing system performs its activity in both male and female gamete sides, leading to homozygous or biallelic plants in the T1 generation. Non-transgenic homozygous or biallelic plants are also potentially obtained in T1 generation, due to meiotic recombination and chromosome assortment. (For ease of identification, we only mapped one pair of homologous chromosomes.) **(D)** The proposed actual working model for the MSC system in Arabidopsis T1 transgenic generation. Due to the limitation of transformation, only heterozygous plants could be obtained in the T1 generation. However, much higher proportion of homozygous or biallelic plants will be obtained in the T2 generation.

As the most powerful genome editing tool, the CRISPR/Cas9 system has been widely used in various species to generate targeted mutations for functional studies. The mutagenesis efficiency is highly dependent on the activity of promoter used for driving *Cas9* expression, directly deciding the original cells generating mutations. In flowering plants, constitutive promoters, such as *35S* and ubiquitin ones, are mostly used to drive *Cas9* expression. Although these types of CRISPR/Cas9 systems can produce heritable gene mutations in T1 plants (Feng et al., 2014), where and what kind of cell the heritable mutation exactly generated is completely unknown. Another shortage for those systems is that variously non-heritable mutations can be created in many somatic cells. To overcome this limitation, germline cell-specific promoters, including both female (egg cell-specific *EC1.2*) and male (pollen-specific *LAT52*), has been used to drive the expression of *Cas9* (Wang et al., 2015; Mao et al., 2016), expecting to obtain genome-edited mutation in gametes exclusively. Thus, subsequent fertilization and following series development will make an individual with unified genotype in the T1 generation. In theory, the T1 plant should be always heterozygous, because only female or male gamete is designed to be edited (**Figures 6A,B**). However, it was reported that homozygous mutants were detected in T1 generation in both systems (Wang et al., 2015; Mao et al., 2016), clearly implying that the real case is not exactly like what it was thought.

To further improve the CRISPR/Cas9 system in Arabidopsis, we selected the experimentally approved meiocyte-specific promoter (*AtDMC1* promoter) to drive *Cas9* expression, forming the MSC system. During plant reproductive development, meiocytes undergo meiosis to generate haploid gametophyte. Each meiocyte eventually forms four daughter cells, harboring only half of the parental genome (Ma, 2006). Because the *AtDMC1* promoter is active in both male and female meiocytes (Klimyuk and Jones, 1997), the MSC system is expected to work in both male and female meiocytes during meiosis, potentially generating male and female haploid daughter cells with mutated gene, respectively. After double fertilization, the combination of a mutated male gamete (sperm) with a mutated female gamete (egg) will form a zygote with a homozygous or biallelic genotype for the targeted gene (**Figure 6C**), leading to the developed individual with identical genotype for all cells. Compared to the egg cell-specific and pollen-specific promoter driving systems, the MSC system is expected to generate a high ratio of homozygous mutants in the T1 generation, because it can simultaneously edit male and female reproductive cells. However, in our studies, including both targeted genes *AtDET2* and *AtDMC1*, we did not observe any individual plant showing mutant phenotypes in T1 generation (**Figures 1C, 3C**). However, DNA sequencing results revealed that the portion of heterozygous plants in T1 generation is very high (**Figure 1D**).

As the T1 plants were obtained by *Agrobacterium*-mediated T-DNA insertion (Clough and Bent, 1998), the time of T-DNA integrated into the plant genome is important for the MSC efficiency. It was revealed that the ovules were the primary targets of *Agrobacterium*-mediated floral dipping transformation in Arabidopsis (Ye et al., 1999). It reasoned that although the MSC system was designed to work in both male and female sides during meiosis, due to the timing of the T-DNA integration, it might only work in female side during T1 generation formation, resulting in the heterozygous T1 plants available only (**Figure 6D**). A previous study has selected a meiocyte-promoter, based on transcriptome data, to drive *Cas9* expression. However, it was shown that homozygous T1 plants were detected (Eid et al., 2016). We thought it might be because the selected promoter is not specific enough for meiosis. By contrast, the *AtDMC1* promoter is experimentally approved to be meiocyte-specific, so we didn’t obtain homozygous plants in T1 generation. As expected, in the T2 generation, the advantage of the MSC system was fully explored (**Figures 1E, 6D**). In addition, as the MSC system exclusively works during meiosis, the generated edited-mutation and the T-DNA inserted fragment could be separated into different haploid daughter cells through meiotic recombination and chromosome assortment. This will eventually lead to the formation of non-transgenic gametes in the T1 generation. Therefore, the combination of both male and female non-transgenic gametes will give rise to the non-transgenic mutations in the T1 generation. Interestingly, a recent study in maize showed that the maize *DMC1* promoter can be used to drive *Cas9* gene expression in callus, consequently generating high genome editing efficiency (Feng et al., 2018). Even so far, the activity of maize *DMC1* promoter has not been shown to be highly restricted to meiocytes or not during plant development, the above study indeed expand the advantage of *DMC1* promoter in genome editing system to crop breeding.

### The MSC System Sufficiently Mediates a Middle Size Deletion Formation

It was generally thought that the CRISPR/Cas9 system utilizes the cellular NHEJ repair pathway to repair the DSBs created by Cas9, consequently generating mutated genes for genetic studies (Shen et al., 2017). However, in most cases, those mutated genes could still make proteins possessing partial functions, making it harder to conclude their exact functions. Therefore, it is ideal to get rid of the full gene from the genome, making completely null mutants. Although it has been reported that CRISPR/Cas9-mediated genome editing system can generate large chromosomal deletion (115–245 kb) at two cleavage sites in rice protoplasts (Zhou et al., 2014), there are relatively few studies focusing on such events in Arabidopsis. The formation of deleted DNA fragment between the two sgRNAs recognizing sites is highly dependent on the coordination of Cas9 cutting time and subsequent DSBs repair. In the MSC system, the Cas9 activity is restricted to meiocytes where meiosis undergoes. During that time (meiosis), homologous chromosome interaction occupies much longer time, potentially making it more possible to obtain the targeted fragment deletion. In our study, taking *AtDET2* as an example, we designed two Cas9 cleavage sites apart ∼500 bp long (**Figure 4A**) for the MSC system testing. Among the obtained 34 T1 plants, it was shown complete removal of the targeted fragment. Thus, our data directly provide the evidence that the MSC system can make middle size deletion at a high efficiency rate. Because the technique for efficient site-directed DNA fragment removal will greatly improve the functional studies for those genes having multiple domains and functional non-coding DNA elements, we strongly recommend to employ the MSC system to take the job.

### The Suicide-MSC System Makes It More Convenient for Studying Genes Involved in Reproductive Development

In Arabidopsis, the CRISPR/Cas9 system is usually delivered to plant cells via *Agrobacterium*-mediated T-DNA transformation, being randomly integrated into the plant genome with different copy numbers. Although the CRISPR/Cas9-mediated mutation can be used for genetic studies to decipher gene’s molecular function, the following functional confirmation via molecular complementary experiments or genetic allelic test would be greatly hampered by the prolonged exist of the CRISPR/Cas9 system. Although it is possible to gain CRISPR/Cas9-free mutated plants by genetic crosses with wild type plants, the success of this strategy is relied on the copy numbers of T-DNA insertion and genetic linkage with the targeted gene. Therefore, it is necessary for molecular functional studies to destroy the CRISPR/Cas9 system after making the targeted gene mutated. In this study, we designed a system containing a sgRNA*^SpCas9^* capable of editing *Cas9* itself, named suicide-MSC system, expecting to destroy the *Cas9* gene while it makes targeted gene mutations.

In this study, taking *AtDET2* as the tested gene once again, we designed the suicide-MSC system to edit *AtDET2* and *Cas9* at the same time (**Figure 5B**). As expected, we obtained heterozygous plants for both *AtDET2* and *Cas9* in the T1 generation (**Figure 5C**), indicating the suicide-MSC system did work in both genes. In addition, the suicide-MSC system can make contributions for keeping mutated genotype uniform for genes whose mutations can only retain in heterozygous types, such as genes involved in reproductive development, including meiosis, anther development, pollen and female gametes development (Ma, 2006). Obviously, the heterozygous plants generated by the CRISPR/Cas9 system cannot be stably inherited, due to Cas9 will continue to edit the wild-type allele, leading to newly mutated allele. Therefore, the suicide-MSC system successfully overcomes the above mentioned limitations, making it promising for application in studies of reproductive genes.

## Materials and Methods

### Plant Materials and *Agrobacterium*-Mediated Transformation

*Arabidopsis thaliana* (Columbia-0 ecotype) were used in this study. All plants were grown in a greenhouse under long-day conditions (16-h light and 8-h dark) at 22°C. Wild-type plants were used for *Agrobacterium*-mediated transformation, in which the floral dipping method was conducted as previously reported (Clough and Bent, 1998). Seeds were harvested from the transformed plants. After gradually dried at 37°C for at least 2 days, the seeds were sterilized with 75% ethanol for 10 min, and further sterilized with 100% ethanol for 30 s. Subsequently, the seeds were plated on 1/2 Murashige and Skoog (MS) medium containing 25 mg/L hygromycin. Two weeks later, positive transgenic seedlings were transplanted to soil for further growth.

### Plasmid Construction

To make a meiocyte-specific promoter driving the *Cas9* gene expression, a 3165 bp long fragment of Pro: *AtDMC1* was cloned from Arabidopsis Columbia-0 genomic DNA, using the specific primers of oLF1464 and oLF1763. Because the restriction enzymes, including *Hind* III, *Nco* I and *Bsa* I, will be used for making constructions, the overlap extension PCR approach was employed to make site-mutagenesis for the above restriction sites in the *AtDMC1* promoter. Firstly, the *AtDMC1* promoter was divided into two fragments, named as P1 and P2. P1 was amplified by PCR using a pair of primers oLF1317 and oLF1318. P2 was amplified using primers oLF1319 and oLF1320. Subsequently, the overlap extension PCR with the primers oLF1317 and oLF1320 was conducted to generate the point mutated *AtDMC1* promoter with destroyed *Bsa* I and *Hind* III sites.

Meanwhile, we amplified the Pro: *U3-*sgRNA fragment from pRGEB31 (Xie et al., 2015) by PCR with a pair of specific primers oLF1585 and oLF1316. The Pro: *U3-*sgRNA fragment and the mutated Pro: *AtDMC1* fragment were linked together by overlap extension PCR using primers oLF1585 and oLF1763. Subsequently, the Pro: *U3-*sgRNA*-*Pro: *AtDMC1* fragment was inserted into pRGEB31 by using *Hind* III and *Nco* I digestion to replace the original Pro: *U3*-sgRNA-Pro: *35S* fragment, forming the Intermediate pMSC plasmid.

To construct the pLFC286, pLFC290, pLFC312, and pLFC371 plasmids, we designed the sgRNAs according to an online tool (see footnote 1). Subsequently, the candidate sgRNA-scaffold-tRNA fragments were subjected to secondary structure analysis using an RNA folding platform^2^. Then the specific primers for assembling fragments of *PTGs* (Polycistronic tRNA-gRNAs) were designed by a previously reported protocol (Xie et al., 2015). The pGTR plasmid (Supplementary Figure S1C) with a gRNA-tRNA fused fragment was used as the template to synthesize *PTGs* harboring sgRNAs. In this study, the *PTGs*, including *PTG1, PTG2, PTG3*, and *PTG4* (Supplementary Table S2), were synthesized by Golden Gate assembly (Supplementary Figure S3) (Engler et al., 2008). The digested *PTGs* by *Fok* I were cloned into the pMSC plasmid, which was digested by *Bsa* I, to give rise to the final pLFC286, pLFC290, pLFC312, and pLFC371 plasmids, respectively. The pLFC286 plasmid harbors sgRNA1*^AtDET2^*. The pLFC371 plasmid has two sgRNAs, sgRNA1*^AtDET2^* and sgRNA2*^AtDET2^*. The pLFC290 contains one sgRNA^AtDMC1^; and the pLFC312 plasmid includes two sgRNAs, sgRNA*^AtDET2^* and sgRNA*^SpCas9^*. All those binary plasmids were used for the *Agrobacterium*-mediated Arabidopsis transformation. The primers used in this study were listed in the Supplementary Table S1.

### Detection of Targeted Gene Mutations

Genomic DNA was extracted from transgenic plants’ leaf by the CTAB method (Rowland and Nguyen, 1993), DNA fragments that surrounding target sites were amplified by PCR with specific primers, the 1,012 bp fragment that covers the target sites of *AtDET2* were amplified by PCR with a pair of primers oLF1852 and oLF1853, the fragment that surrounding the *AtDMC1* target sites were amplified with the primers oLF1870 and oLF1871, which is 985 bp, in a similar way, the sequence surrounding the *SpCas9* target sites were amplified with primers oLF1868 and oLF1869, and then we submitted those PCR products for direct sequencing with primers oLF1852, oLF1870, and oLF1868 located within the PCR products, and then the sequencing results aligning with wild type control sequence and screened mutations. To further identify the mutants’ genotypes, we also purified the PCR products by a toolkit (Axygen PCR clear toolkit) and cloned them into the pEASY-Blunt vector (TransGen Biotech, Simple Cloning Kit, CB111-01), then transformed it into *E. coli* DH5α, submitted the individual positive clones for Sanger sequencing with primers oLF1072 located in pEASY-Blunt vector. Detection of targeted gene mutations was performed with leaf samples. Details of all the primers are shown in Supplementary Table S1.

## Author Contributions

PX, HS, and WC performed the experiments and analyzed the data. PL designed the study and supervised all of the work. PL and PX wrote the manuscript. All authors read and approved the final manuscript.

## Conflict of Interest Statement

The authors declare that the research was conducted in the absence of any commercial or financial relationships that could be construed as a potential conflict of interest.

## Footnotes

1 http://cbi.hzau.edu.cn/cgi-bin/CRISPR

2 http://rna.tbi.univie.ac.at/cgi-bin/RNAWebSuite/RNAfold.cgi
