# Supplementary material for: The Application of a Meiocyte-Specific CRISPR/Cas9 (MSC) System and a Suicide-MSC System in Generating Inheritable and Stable Mutations in Arabidopsis
Source: Front Plant Sci. 2018 Jul 13;9:1007. doi: 10.3389/fpls.2018.01007 (PMC6055057; doi:10.3389/fpls.2018.01007)
Supplement: Supplementary file 1 [file Presentation_1.pdf]

Figure S1

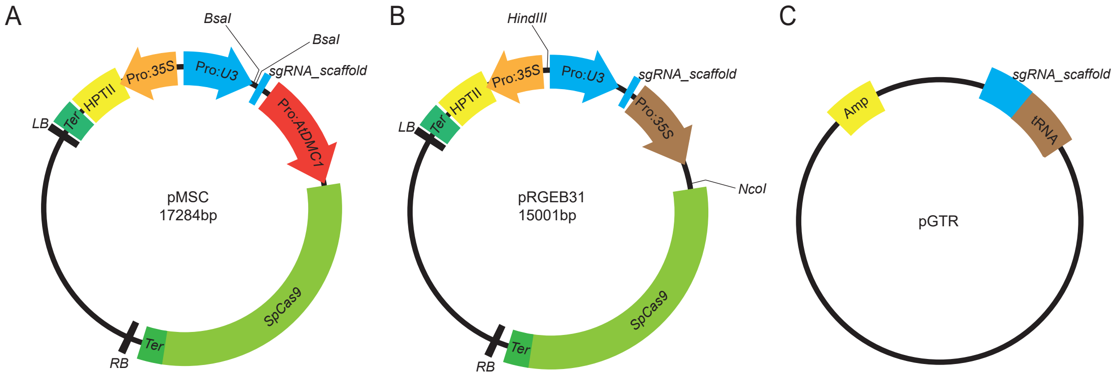
A
BsaI
BsaI
sgRNA_scaffold
Pro:35S
Pro:U3
HPTII
Ter
LB
Pro:AtDMC1
pMSC
17284bp
SpCas9
Ter
RB
B
HindIII
sgRNA_scaffold
Pro:35S
Pro:U3
HPTII
Ter
LB
Pro:35S
NcoI
pRGEB31
15001bp
SpCas9
Ter
RB
C
sgRNA_scaffold
Amp
tRNA
pGTR

Figure S2

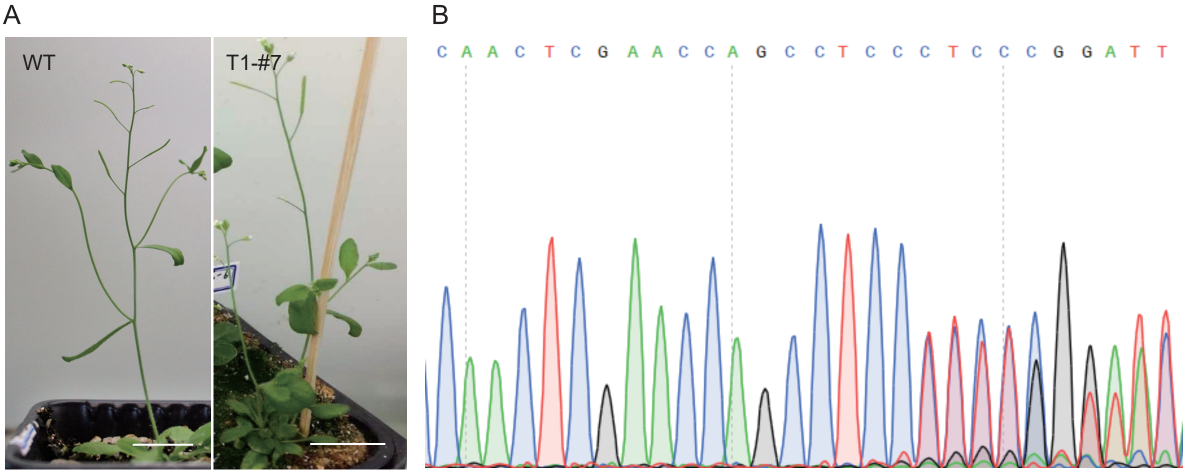

Figure S3

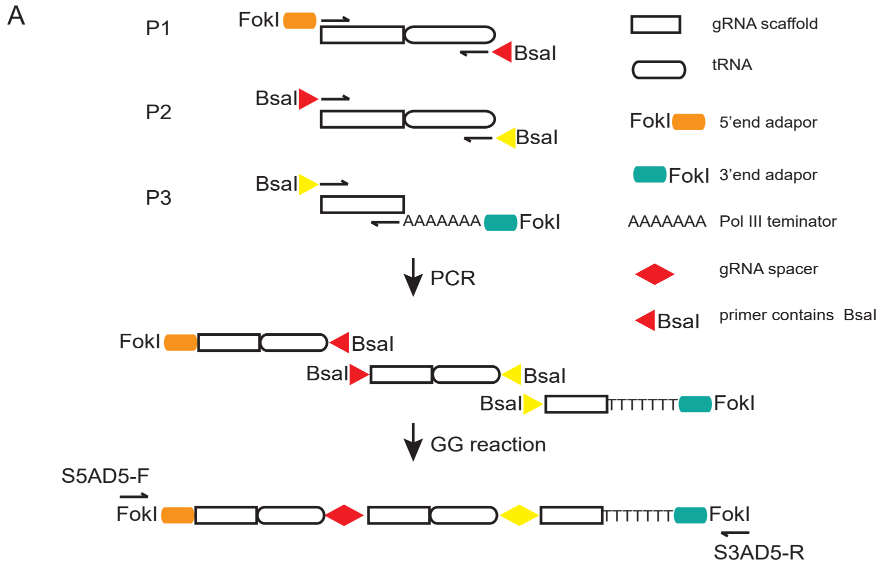

B

	Primers/PTG1 for pLFC286		Primers/PTG2 for pLFC290		Primers/PTG3 for pLFC312		Primers/PTG4 for pLFC371	
P1	oLF258	oLF1772	oLF258	oLF1794	oLF258	oLF1817	oLF258	oLF1774
P2	oLF1773	oLF259	oLF1795	oLF259	oLF1818	oLF1772	oLF1775	oLF1772
P3					oLF1773	oLF259	oLF1773	oLF259

Figure S4

A

sgRNA^{DET2} T1

```
WT   AACTCGAACCAGCCTCC TCTCGG
     AACTCGAACCAGCCTCC TCTCGG
#14  AACTCGAACCAGCCTCC TCTCGG
     AACTCGAACCAGCCTCC TCTCGG
#15  AACTCGAACCAGCCTCC TCTCGG
     AACTCGAACCAGCCTCC TCTCGG
#16  AACTCGAACCAGCCT---------   -27bp
     AACTCGAACCAGCCTCC TCTCGG
#17  AACTCGAACCAGCCTCC -CTCGG   -T
     AACTCGAACCAGCCTCC TCTCGG
#18  AACTCGAACCAGCCTCC TCTCGG
     AACTCGAACCAGCCTCC TCTCGG
#19  AACTCGAACCAGCCTCCCTCTCGG   +C
     AACTCGAACCAGCCTCC TCTCGG
#20  AACTCGAACCAGCCTCC TCTCGG
     AACTCGAACCAGCCTCC TCTCGG
#21  AACTCGAACCAGCCTCC --TCGG   -TC
     AACTCGAACCAGCCTCC TCTCGG
#22  AACTCGAACCAGCCTCC TCTCGG
     AACTCGAACCAGCCTCC TCTCGG
#23  AACTCGAACCAGCCT-- --TCGG   -CTTC
     AACTCGAACCAGCCTCC TCTCGG
#24  AACTCGAACCAGCCTC- TCTCGG   -C
     AACTCGAACCAGCCTCC TCTCGG
#25  AACTCGAACCAGCCTCC TCTCGG
     AACTCGAACCAGCCTCC TCTCGG
```

B

sgRNA^{DET2} T2

```
WT  AACTCGAACCAGCCTCC TCTCGG
    AACTCGAACCAGCCTCC TCTCGG
#13 AACTCGAACCAGCCTC- TCTCGG   -C
    AACTCGAACCAGCCTC- TCTCGG   -C
#14 AACTCGAACCAGCCTC- TCTCGG   -C
    AACTCGAACCAGCCTC- TCTCGG   -C
#15 AACTCGAACCAGCCTCCCTCTCGG   +C
    AACTCGAACCAGCCTCCCTCTCGG   +C
#16 AACTCGAACCAGCCTCCCTCTCGG   +C
    AACTCGAACCAGCCTCCCTCTCGG   +C
#17 AACTCGAACCAGCCTCCTTCTCGG   +T
    AACTCGAACCAGCCTCCTTCTCGG   +T
#18 AACTCGAACCAGCCTCCCTCTCGG   +C
    AACTCGAACCAGCCTCCCTCTCGG   +C
#50 AACTCGAACCAGCCTCCCTCTCGG   +C
    AACTCGAACCAGCCTCC TCTCGG
#51 AACTCGAACCAGCCTCCCTCTCGG   +C
    AACTCGAACCAGCCTCC TCTCGG
#52 AACTCGAACCAGCCTCCCTCTCGG   +C
    AACTCGAACCAGCCTCC TCTCGG
#53 AACTCGAACCAGC-TCC TCTCGG   -C
    AACTCGAACCAGCCTCC TCTCGG
#54 AACTCGAACCAGCCTCCCTCTCGG   +C
    AACTCGAACCAGCCTCC TCTCGG
```

C

sgRNA^{DMC1} T1

```
WT  ATGCATCATGAGACCAT TGCAGG
    ATGCATCATGAGACCAT TGCAGG
#10 ATGCATCATGAGACCA- ----GG  -TTGCA
    ATGCATCATGAGACCAT TGCAGG
#11 ATGCATCATGAGACCA- -----G  -TTGCAG
    ATGCATCATGAGACCAT TGCAGG
#12 ATGCATCATGAGACCAT TGCAGG
    ATGCATCATGAGACCAT TGCAGG
#13 ATGCATCATGAGACCATGTGCAGG  +G
    ATGCATCATGAGACCAT TGCAGG
#14 ATGCATCATGAGACCAT -GCAGG  -T
    ATGCATCATGAGACCAT TGCAGG
#15 ATGCATCATGAGACCAT -GCAGG  -T
    ATGCATCATGAGACCAT TGCAGG
```

Figure S5

A

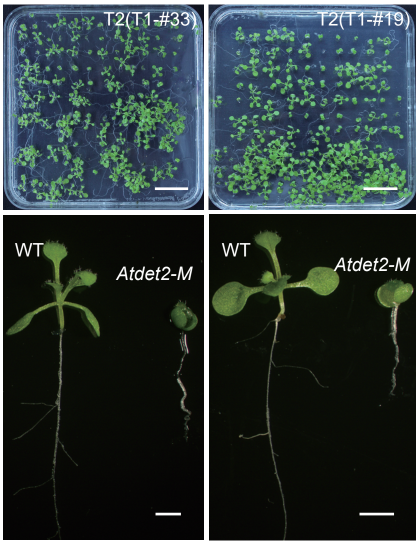

B

```
pLFC286 T2(T1-33)
WT    AACTCGAACCAGCCTCC TCTCGG
      AACTCGAACCAGCCTCC TCTCGG
#1    AACTCGAACCAGCCTCCCTCTCGG   +C
      AACTCGAACCAGCCTCC TCTCGG
#2    AACTCGAACCAGCCTCCCTCTCGG   +C
      AACTCGAACCAGCCTCC TCTCGG
#3    AACTCGAACCAGCCTCCCTCTCGG   +C
      AACTCGAACCAGCCTCC TCTCGG
#4    AACTCGAACCAGCCTCCCTCTCGG   +C
      AACTCGAACCAGCCTCC TCTCGG
#5    AACTCGAACCAGCCTCCCTCTCGG   +C
      AACTCGAACCAGCCTCCCTCTCGG   +C
#6    AACTCGAACCAGCCTCCCTCTCGG   +C
      AACTCGAACCAGCCTCC TCTCGG
#7    AACTCGAACCAGCCTCCCTCTCGG   +C
      AACTCGAACCAGCCTCC TCTCGG
#8    AACTCGAACCAGCCTCCCTCTCGG   +C
      AACTCGAACCAGCCTCC TCTCGG
#9    AACTCGAACCAGCCTCCCTCTCGG   +C
      AACTCGAACCAGCCTCC TCTCGG
#10   AACTCGAACCAGCCTCCCTCTCGG   +C
      AACTCGAACCAGCCTCC TCTCGG
#11   AACTCGAACCAGCCTCCCTCTCGG   +C
      AACTCGAACCAGCCTCC TCTCGG
#12   AACTCGAACCAGCCTCCCTCTCGG   +C
      AACTCGAACCAGCCTCCCTCTCGG   +C
#13   AACTCGAACCAGCCTCCCTCTCGG   +C
      AACTCGAACCAGCCTCC TCTCGG
#14   AACTCGAACCAGCCTCCCTCTCGG   +C
      AACTCGAACCAGCCTCCCTCTCGG   +C
#15   AACTCGAACCAGCCTCCCTCTCGG   +C
      AACTCGAACCAGCCTCCCTCTCGG   +C
#16   AACTCGAACCAGCCTCCCTCTCGG   +C
      AACTCGAACCAGCCTCCCTCTCGG   +C
#20   AACTCGAACCAGCCTCCCTCTCGG   +C
      AACTCGAACCAGCCTCCCTCTCGG   +C
#21   AACTCGAACCAGCCTCCTTCTCGG   +T
      AACTCGAACCAGCCTCC TCTCGG
#23   AACTCGAACCAGCCTCCCTCTCGG   +C
      AACTCGAACCAGCCTCC TCTCGG
#24   AACTCGAACCAGC---- TCTCGG   -CTCC
      AACTCGAACCAGCCTCC TCTCGG
#25   AACTCGAACCAGCCTCCCTCTCGG   +C
      AACTCGAACCAGCCTCC TCTCGG
#27   AACTCGAACCAGCCTCCCTCTCGG   +C
      AACTCGAACCAGCCTCCCTCTCGG   +C
#28   AACTCGAACCAGCCTCCCTCTCGG   +C
      AACTCGAACCAGCCTCC TCTCGG
#31   AACTCGAACCAGCCTCCCTCTCGG   +C
      AACTCGAACCAGCCTCC TCTCGG
#32   AACTCGAACCAGCCTCCCTCTCGG   +C
      AACTCGAACCAGCCTCCCTCTCGG   +C
#33   AACTCGAACCAGCCTCCCTCTCGG   +C
      AACTCGAACCAGCCTCC TCTCGG
#34   AACTCGAACCAGCCTCCCTCTCGG   +C
      AACTCGAACCAGCCTCCCTCTCGG   +C
#35   AACTCGAACCAGCCTCCCTCTCGG   +C
      AACTCGAACCAGCCTCC TCTCGG
#36   AACTCGAACCAGCCTCCCTCTCGG   +C
      AACTCGAACCAGCCTCCCTCTCGG   +C
#38   AACTCGAACCAGCCTCCCTCTCGG   +C
      AACTCGAACCAGCCTCCCTCTCGG   +C
```

```
pLFC286 T2(T1-#19)
WT    AACTCGAACCAGCCTCC TCTCGG
      AACTCGAACCAGCCTCC TCTCGG
#1    AACTCGAACCAGCCTCC -CTCGG   -T
      AACTCGAACCAGCCTCC TCTCGG
#2    AACTCGAACCAGCCTCCCTCTCGG   +C
      AACTCGAACCAGCCTCCCTCTCGG   +C
#3    AACTCGAACCAGCCTCCCTCTCGG   +C
      AACTCGAACCAGCCTCCCTCTCGG   +C
#4    AACTCGAACCAGCCTCCCTCTCGG   +C
      AACTCGAACCAGCCTCCCTCTCGG   +C
#5    AACTCGAACCAGC---- TCTCGG   -CTCC
      AACTCGAACCAGCCTCC TCTCGG
#6    AACTCGAACCAGC---- TCTCGG   -CTCC
      AACTCGAACCAGCCTCCTTCTCGG   +T
#7    AACTCGAACCAGCCTCCCTCTCGG   +C
      AACTCGAACCAGCCTCCCTCTCGG   +C
#8    AACTCGAACCAGCCTCCCTCTCGG   +C
      AACTCGAACCAGCCTCC TCTCGG
#9    AACTCGAACCAGCCTCCCTCTCGG   +C
      AACTCGAACCAGCCTCCCTCTCGG   +C
#10   AACTCGAACCAGCCTCCCTCTCGG   +C
      AACTCGAACCAGCCTCC TCTCGG
#11   AACTCGAACCAGCCTCCCTCTCGG   +C
      AACTCGAACCAGCCTCC TCTCGG
#12   AACTCGAACCAGCCTCCCTCTCGG   +C
      AACTCGAACCAGCCTCC TCTCGG
#13   AACTCGAACCAGCCTCCCTCTCGG   +C
      AACTCGAACCAGCCTCC TCTCGG
#15   AACTCGAACCAGC---- TCTCGG   -CTCC
      AACTCGAACCAGCCTCCTTCTCGG   +T
#16   AACTCGAACCAGCCTCCCTCTCGG   +C
      AACTCGAACCAGCCTCCCTCTCGG   +C
#17   AACTCGAACCAGCCTCCCTCTCGG   +C
      AACTCGAACCAGCCTCC TCTCGG
#18   AACTCGAACCAGCCTCCCTCTCGG   +C
      AACTCGAACCAGCCTCCCTCTCGG   +C
#19   AACTCGAACCAGC---- TCTCGG   -CTCC
      AACTCGAACCAGCCTCCTTCTCGG   +T
#20   AACTCGAACCAGCCTCCCTCTCGG   +C
      AACTCGAACCAGCCTCCCTCTCGG   +C
#21   AACTCGAACCAGCCTCCCTCTCGG   +C
      AACTCGAACCAGCCTCCCTCTCGG   +C
#22   AACTCGAACCAGCCTCCCTCTCGG   +C
      AACTCGAACCAGCCTCCCTCTCGG   +C
#23   AACTCGAACCAGCCTCCCTCTCGG   +C
      AACTCGAACCAGCCTCCCTCTCGG   +C
#24   AACTCGAACCAGC---- TCTCGG   -CTCC
      AACTCGAACCAGCCTCCTTCTCGG   +T
#25   AACTCGAACCAGCCTCCCTCTCGG   +C
      AACTCGAACCAGCCTCC TCTCGG
#26   AACTCGAACCAGCCTCCCTCTCGG   +C
      AACTCGAACCAGCCTCC TCTCGG
#28   AACTCGAACCAGCCTCCCTCTCGG   +C
      AACTCGAACCAGCCTCC TCTCGG
#29   AACTCGAACCAGCCTCCCTCTCGG   +C
      AACTCGAACCAGCCTCC TCTCGG
#30   AACTCGAACCAGCCTCCCTCTCGG   +C
      AACTCGAACCAGCCTCC TCTCGG
#31   AACTCGAACCAGCCTCCCTCTCGG   +C
      AACTCGAACCAGCCTCCCTCTCGG   +C
#32   AACTCGAACCAGCCTCCCTCTCGG   +C
      AACTCGAACCAGCCTCCCTCTCGG   +C
#33   AACTCGAACCAGCCTCCCTCTCGG   +C
      AACTCGAACCAGCCTCC TCTCGG
#34   AACTCGAACCAGCCTCCCTCTCGG   +C
      AACTCGAACCAGCCTCC TCTCGG
#35   AACTCGAACCAGCCTCCCTCTCGG   +C
      AACTCGAACCAGCCTCCCTCTCGG   +C
#36   AACTCGAACCAGCCTCCCTCTCGG   +C
      AACTCGAACCAGCCTCCCTCTCGG   +C
#37   AACTCGAACCAGCCTCCCTCTCGG   +C
      AACTCGAACCAGCCTCCCTCTCGG   +C
#38   AACTCGAACCAGCCTCCCTCTCGG   +C
      AACTCGAACCAGCCTCCCTCTCGG   +C
#39   AACTCGAACCAGCCTCCCTCTCGG   +C
      AACTCGAACCAGCCTCCCTCTCGG   +C
#40   AACTCGAACCAGC---- TCTCGG   -CTCC
      AACTCGAACCAGC---- TCTCGG   -CTCC
#41   AACTCGAACCAGCCTCCCTCTCGG   +C
      AACTCGAACCAGCCTCCCTCTCGG   +C
#42   AACTCGAACCAGCCTCCCTCTCGG   +C
      AACTCGAACCAGCCTCC TCTCGG
```

C

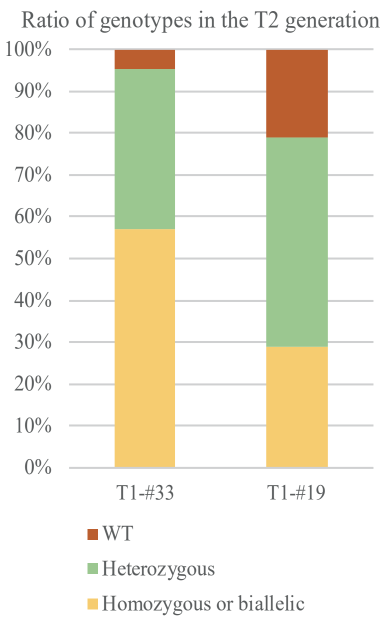

Supplementary materials

Table S1. The list of primers used in this study

Table S2. Sequences of all PTGs (Polycistronic tRNA-gRNAs)

Table S3. Statistical analysis of T1 mutants transformed pLFC312 plasmid

Table S4. Phenotypic analysis of the T2 generation from the T1 line #7.

Figure S1. The maps of plasmids used in this study. (A) The map of the pMSC vector**.** **(B)** The map of the pRGEB31 vector. **(C)** The map of the pGTR vector. The plasmid with a gRNA-tRNA fused fragment was used as the template to synthesize PTGs.

Figure S2. The phenotype and genotype analysis of the T1 plant line #7 obtained from the transformation of pLFC286 for editing *AtDET2*. (A) Phenotypes of wild type plant and the transgenic line #7 in T1 generation. Bar = 1cm. **(B)** The sequencing result of the line #7 indicated it was a heterozygous plant, results of monoclonal sequencing also indicated that.

Figure S3. Synthesis of PTGs (Polycistronic tRNA-gRNAs) and primers for amplifying fragments for PTGs assembly.

Figure S4. Genotyping results of *Atdet2 and Atdmc1* mutants generated by the MSC system in the T1 and T2 generations. Only show the edited line.

Figure S5. Genotypic and phenotypic analyses of progenies of T1 line #19 and #33 generated by the MSC system. (A) Phenotypes of the population of T1 line #19 and #33, Plants have grown for 14 days on 1/2 Murashige and Skoog (MS) medium before photographing. Up figures, bar = 2cm. Down figures, bar = 1mm. **(B)** Sequencing results of T2 generation. Only show the mutant. **(C)** Ratio of genotypes in the T2 generation. In the progenies of T1 line #19 and #33, the homozygous or biallelic account for 29% and 57%, respectively.

Table S1 The list of primers used in this study

Primer name	Sequence 5′-3′
oLF1464	AGGTCCGCGTTAACACCGTTTATATG
oLF1465	TATATTGTGAGACTTGATCAAGTTCAAG
oLF1588	GGCTCTAGATTTCTCGCTCTAAGA
oLF1751	TGGGGCCATTCTAGATTTCTCGCTCTAAGAGTCTCTAAACTTGGAAGAGTGA
oLF1763	ATCCCATGGTTTCTCGCTCTAAGAGTCTCTAAACTTGGAAGAGTGA
oLF1316	CATATAAACGGTGTTAACGCGGACCTGCAGGCATGCA
oLF1317	GCGTTAACACCGTTTATATG
oLF1318	GTGAGACTTGATCAAGTTCAAG
oLF1319	CTTGAACTTGATCAAGTCTCAC
oLF1320	GGCACTAGTTTTCTCGCTCTAAGA
oLF1585	GATTACGCCAAGCTTAAGGAATCTTTAAACATACGAA
oLF1072	TGTAAAACGACGTCCAGT
oLF1073	CAGGAAACAGCTATGACC
oLF1772	CGGGTCTCACTGGTTCGAGTTTGCACCAGCCGGG
oLF1773	TAGGTCTCCCCAGCCTCCTCTGTTTTAGAGCTAGAA
oLF1774	CGGGTCTCAGCTCACTCTCCTTGCACCAGCCGGG
oLF1775	TAGGTCTCCGAGCCACAAGGTGTTTTAGAGCTAGAA
oLF1792	CGGGTCTCACTCGTTGAGCGTGTGCACCAGCCGGG
oLF1793	TAGGTCTCCCGAGCTGCATCGTTTTAGAGCTAGAA
oLF1794	CGGGTCTCACTCATGATGCATTGCACCAGCCGGG
oLF1795	TAGGTCTCCTGAGACCATTGCGTTTTAGAGCTAGAA
oLF1817	CGGGTCTCATCTTCCAGTCTGTGCACCAGCCGGG
oLF1818	TAGGTCTCCAAGAGTCCTTCCGTTTTAGAGCTAGAA
oLF1819	CGGGTCTCATTGTCGGGGTTCTGCACCAGCCGGG
oLF1820	TAGGTCTCCACAACAGCGACGGTTTTAGAGCTAGAA
oLF258	CGGGTCTCAGGCAGGATGGGCAGTCTGGGCAACAAAGCACCAGTGG
oLF259	TAGGTCTCCAAACGGATGAGCGACAGCAAACAAAAAAAAAAGCACCGACTCG
oLF260	CGGGTCTCAGGCAGGATGGGCAGTCTGGGCA
oLF261	TAGGTCTCCAAACGGATGAGCGACAGCAAAC
oLF262	AGTACCACCTCGGCTATCCACA
oLF263	GGACCTGCAGGCATGCACGCGCTAAAAACGGACTAGC
oLF1852	CCGGTTACTGGAAATTTTGACAAAG
oLF1853	TTCTGCCCTTATTAGAGAGATCTGA
oLF1870	GCAACAACAGATGGAAACGTT
oLF1871	TCTTAGAGCGAGAAAATGATGGC
oLF1868	AGAAGTACAGCATCGGCCTGGAC
oLF1869	TGGTGATCTCGGTGTTCACTCTCAGGA

Table S2 Sequences of all PTGs (Polycistronic tRNA-gRNAs)

Architecture	Sequences (5′- 3′)
**PTG1**	gatccgtggcaacaaagcaccagtggtctagtggtagaatagtaccctgccacg gtacagacccgggttcgattcccggctggtgcaaactcgaaccagcctcctctgtt ttagagctagaaatagcaagttaaaataaggctagtccgttatcaacttgaaaaa gtggcaccgagtcggtgcttttttt
**PTG2**	gatccgtggcaacaaagcaccagtggtctagtggtagaatagtaccctgccacg gtacagacccgggttcgattcccggctggtgcaatgcatcatgagaccattgcgtt ttagagctagaaatagcaagttaaaataaggctagtccgttatcaacttgaaaaa gtggcaccgagtcggtgcttttttt
**PTG3**	gatccgtggcaacaaagcaccagtggtctagtggtagaatagtaccctgccacg gtacagacccgggttcgattcccggctggtgcacagactggaagagtccttccgt tttagagctagaaatagcaagttaaaataaggctagtccgttatcaacttgaaaaa gtggcaccgagtcggtgaacaaagcaccagtggtctagtggtagaatagtaccc tgccacggtacagacccgggttcgattcccggctggtgcaaactcgaaccagcc tcctctgttttagagctagaaatagcaagttaaaataaggctagtccgttatcaactt gaaaaagtggcaccgagtcggtgcttttttt
**PTG4**	gatccgtggcaacaaagcaccagtggtctagtggtagaatagtaccctgccacg gtacagacccgggttcgattcccggctggtgcaaactcgaaccagcctcctctgtt ttagagctagaaatagcaagttaaaataaggctagtccgttatcaacttgaaaaa gtggcaccgagtcggtgcaacaaagcaccagtggtctagtggtagaatagtacc ctgccacggtacagacccgggttcgattcccggctggtgcaaggagagtgagc cacaaggtgttttagagctagaaatagcaagttaaaataaggctagtccgttatca acttgaaaaagtggcaccgagtcggtgcttttttt

Table S3 Statistical analysis of T1 mutants transformed pLFC312 plasmid

	**Number of sequencing positive transgenic plants**	***Number of DET2 mutation***	***Percentage of DET2 mutation***	***Number of SpCas9 mutation***	***Percentage of SpCas9 mutation***	***Percentage of DET2 SpCas9 mutation***
**pLFC312**	6	4	67%	4	67%	67%

Table S4 Phenotypic analysis of the T2 generation from the T1 line #7 for editing *AtDET2*.

Groups	Number of plants	Phenotypes of plants	
		Mutant phenotype	Normal-like phenotype
1	#1-#103	#1-#49	#50-#103
2	#104-#184	#104-#128	#129-#184
3	#185-#257	#185-#206	#207-#257
